# Implication of Chronotherapy in mouse models of Alzheimer’s Disease

**DOI:** 10.1002/alz.094823

**Published:** 2025-01-09

**Authors:** Yu Shi, Ricardo Salinas, William McRoberts, Stephen Rozen, Jordan Swint, George S Bloom, Ali Güler

**Affiliations:** ^1^ University of Virginia, Charlottesville, VA USA

## Abstract

**Background:**

Sleep disturbances are commonly observed in early‐stage Alzheimer’s Disease (AD) patients and correlate with their cognitive performance over time. Sleep in mammals is governed by the central circadian system, which responds to environmental cues like light and aligns with daily routines such as eating.

**Method:**

In this study, we developed a regimen with a shortened light phase (LD = 8:16), time‐restricted feeding, and exercise (LiFE) to strengthen the circadian system and enhance sleep quality in 5xFAD and 5xFAD‐PS19 AD mice.

**Result:**

This approach enhanced cognitive performance in Morris water maze and novel object recognition tests. The LiFE treatment did not reduce amyloid plaques or tau phosphorylation.

**Conclusion:**

Our study shows that, independent of AD histopathological changes, LiFE chronotherapy enhances cognition in AD model mice, with the potential to improve cognition in human AD patients. We are currently exploring the neural mechanisms and biological processes impacted by the LiFE treatment.

